# Complete removal of heart-compressing large mediastinal lipoma : a case report

**DOI:** 10.1186/1749-8090-5-48

**Published:** 2010-06-03

**Authors:** Noritoshi Minematsu, Naoki Minato, Keiji Kamohara, Takeshi Hakuba

**Affiliations:** 1 Department of Thoracic and Cardiovascular Surgery, Fukuoka Tokushukai Hospital, 4-5 Sukukita, Kasuga City, Fukuoka, 816-0864, Japan; 2 Department of Thoracic and Cardiovascular Surgery, Kansai Medical University, Japan

## Abstract

An 83-year-old man presented with worsening of respiratory discomfort and underwent close examination, which revealed a large mediastinal lipoma measuring 15 × 10 cm. The patient showed heart failure symptoms due to heart compression by tumor. The tumor was completely removed safely and reliably by cutting the ascending aorta, main pulmonary artery and superior vena cava. Although preoperative examination could not determine whether the tumor was lipoma or liposarcoma, we selected an invasive surgical therapy because neither radiation therapy nor chemotherapy was considered effective for either type of tumor. We report here a very rare case of heart-compressing mediastinal tumor.

## Introduction

Mediastinal lipoma is a rare tumor of the mediastinum and causes few clinical symptoms [1]. Even if diagnosed pathologically as benign, mediastinal lipoma causing clinical symptoms is considered clinically malignant. We achieved complete removal of a 15 × 10 cm large mediastinal lipoma by cutting the ascending aorta, main pulmonary artery and superior vena cava in a patient who developed heart failure due to heart compression by the tumor. Tumor removal using this approach is rare and is therefore reported here.

## Case report

**Figure 1:**
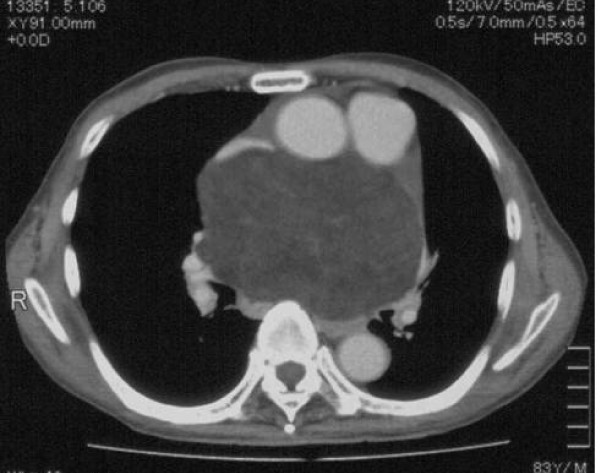
**Computed tomography showed a large tumor behind the ascending aorta and pulmonary artery and compressing the right and left atria**.

An 83-year-old man showed sinus rhythm on echocardiography, but occasionally showed paroxysmal atrial fibrillation suspected to be due to left atrial compression. With respiratory discomfort worsened to NYHA III, the patient underwent close examination by computed tomography and was found to have a large tumor (15 × 10 cm) behind the ascending aorta and pulmonary artery and compressing the right and left atria (Fig 1). The tumor was homogenous, immediately enhanced with contrast medium, rich in fat and showed no apparent invasion into surrounding tissues, suggesting that the tumor was lipoma. All tumor markers, except SCC with a high value of 9.3, showed normal values. Ultrasound cardiography (UCG) demonstrated favorable cardiac function (EF: 70.0%) without asynergy, but revealed mitral regurgitation of III degree due to deformation of the left atrium caused by the tumor. No apparent tumor invasion into the heart was observed on UCG. Cardiac catheterization revealed compression of the left main trunk by the tumor, but no significant coronary stenosis due to arteriosclerosis. A feeding vessel extending from the sinus node artery into the tumor was observed.

Due to advanced age, the patient was considered at high risk, with a EuroSCORE of 8. However, since preoperative imaging suggested a benign tumor with no invasion to surrounding tissues, we judged that surgical resection would improve clinical symptoms and considered the patient eligible for surgery.

## Surgery

**Figure 2:**
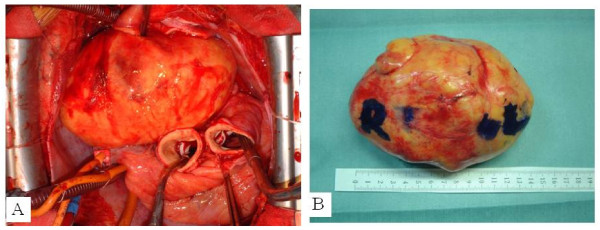
**(A) Intraoperative photograph and (B) surgical specimen showed a giant tumor located within the middle and posterior mediastinum**.

The surgical approach was achieved by median sternotomy. Accumulation of about 40 ml of serous pericardial fluid was found; cytology of the accumulated pericardial fluid showed no tumorous change. The right end of the tumor compressed the superior vena cava from its posterior aspect. The tumor was then detached from the superior vena cava. With no invasion into surrounding tissues, the tumor was considered detachable and removable. Since the tumor was located behind the superior vena cava, ascending aorta and main pulmonary artery, we judged it necessary to cut these vessels to obtain a good surgical field. Extracorporeal circulation was started by transmitting blood to the right femoral artery and draining blood from the superior and inferior vena cava, ascending aorta and main pulmonary artery were cut. The tumor was encapsulated with a thin membrane and showed no invasion into surrounding tissues. A tight adhesion was observed in an extra-pericardial mediastinal fat tissue in the superior-posterior portion of the bifurcation of the right pulmonary artery. Except this portion, the tumor was relatively easily detached from surrounding tissues. A solid tumor encapsulated with a thin membrane was completely removed as a mass (Fig 2A). The tumor weighed 500 g (Fig 2B). The cut superior vena cava, ascending aorta and main pulmonary artery were reanastomosed. Extracorporeal circulation was discontinued without difficulty.

## Pathology

### Macroscopic findings

**Figure 3:**
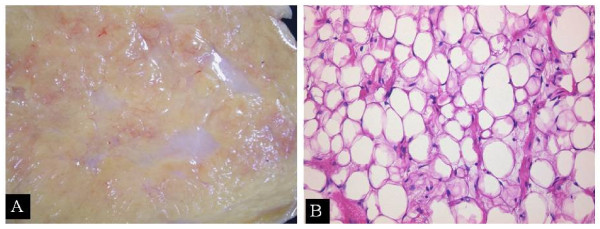
**(A) The tumor showed a multilocular structure with fibrous septa and (B) the majority of cells were differentiated with low cellular density and poor vascular proliferation**.

The tumor was mature, 10 × 15 cm in size, filled with fat, and showed a clear margin. Its surface was smooth and covered by a thin membrane. The cut surface of the tumor showed a multilocular structure with fibrous septa (Fig 3A).

### Microscopic findings

Some tumor cells resembled liposarcoma cells and were variable in size. Unlike liposarcoma, however, the majority of cells were differentiated with low cellular density and poor vascular proliferation (Fig 3B). With the absence of lipoblast characteristic of liposarcoma and abundance of mature fat droplets, the tumor was diagnosed as lipoma.

## Postoperative course

Although the patient was in advance age and thus was hospitalized for a relatively long period of time, he recovered and was discharged from hospital with no serious complications such as quadriplegia.

## Discussion and Conclusions

Lipoma can occur in any soft tissues but is uncommon in the mediastinum, only accounting for 1.6-2.3% of all primary mediastinal tumors [2]. Most cases of mediastinal lipoma occur in the anterior mediastinum. Mediastinal lipoma causes superior vena cava syndrome or Horner's syndrome due to tumor mass effect, spinal nerve paralysis, swallowing disorder due to esophageal compression, respiratory discomfort and arrhythmia [3]. Therefore, although lipoma is histologically diagnosed as a benign tumor and grows slowly, those growing and causing clinical symptoms should be considered clinically malignant and subjected to surgical procedures for complete removal.

Although the patient was in advanced age and considered at high risk, we judged that the patient was eligible for surgical therapy because he showed heart failure symptoms due to heart compression by tumor. We also considered radiation therapy and chemotherapy as alternative options; however, we found a number of reports skeptical about the effectiveness of these therapies and thus considered them to be less effective.

For the surgical approach, thoracoscopic tumor excision has recently been reported. However, since the tumor was large (10 × 15 cm) and adjacent to the posterior aspect of the left atrium, we considered it difficult to perform thoracoscopic surgery due to high risk of bleeding [4]. There also are several case reports of liposarcoma developing after resection of mediastinal lipoma. However, in these cases, although the tumors were pathologically diagnosed as lipoma at the initial surgery, they actually might have been well-differentiated liposarcoma with histological characteristics similar to those of lipoma. In the present case, we made a preoperative diagnosis of lipoma, but could not rule out the possibility of liposarcoma. Well-differentiated liposarcoma has a high local recurrence rate (53%) and a strong tendency toward local recurrence following incomplete resection (local recurrence rate following complete resection: 30%) [5]. Therefore, complete tumor removal was essential, and the thoracoscopic approach was considered inadequate. Although an invasive approach, we chose to perform surgery under direct vision and extracorporeal circulation in consideration of safety and reliability.

We encountered an aged patient with heart failure symptoms of NYHA III who was found to have a large mediastinal tumor and obtained a favorable outcome through complete removal of the tumor after cutting of the ascending aorta, main pulmonary artery and superior vena cava. Although we were aware of the high risk of surgery, we selected an invasive treatment option in consideration of the need for a good surgical field due to the large tumor and for complete removal of the tumor due to high possibility of recurrence. The selected treatment strate allowed the patient to return to his daily activities without recurrence for at least 18 mon postoperation.

## Competing interests

The authors declare that they have no competing interests.

## Authors' contributions

MN was the primary caregiver for this patient and reviewed the manuscript. KK and TH also cared for this patient. NM performed data collection and drafted the manuscript. All authors read and approved the final manuscript.

## Consent

Written informed consent was obtained from the patient for publication of this case report and any accompanying images. A copy of the written consent is available for review by the Editor-in-Chief of this journal.
